# Readmissions, revisions, and mortality after treatment for proximal humeral fractures in three large states

**DOI:** 10.1186/s12891-019-2812-9

**Published:** 2019-09-11

**Authors:** Dominique I. Dabija, Hongshu Guan, Andrew Neviaser, Nitin B. Jain

**Affiliations:** 1 Vanderbilt University School of Medicine, Vanderbilt University, Nashville, TN USA; 2 Department of Medicine, Brigham and Women’s Hospital, Boston, MA USA; 3 Department of Orthopaedics, Ohio State University, Columbus, OH USA; 4 Department of Physical Medicine and Rehabilitation, Vanderbilt University Medical Center, 2201 Children’s Way, Suite 1318, Nashville, TN 37212 USA; 5 Department of Orthopaedics and Rehabilitation, Vanderbilt University Medical Center, Nashville, TN USA

**Keywords:** Proximal humeral fractures, Open reduction and internal fixation, Arthroplasty

## Abstract

**Background:**

Proximal humeral fractures can be treated non-operatively or operatively with open reduction and internal fixation (ORIF) and arthroplasty. Our objective was to assess practice patterns for operative and non-operative treatment of proximal humeral fractures. We also report on complications, readmissions, in-hospital mortality, and need for surgery after initial treatment of proximal humeral fractures in California, Florida, and New York.

**Methods:**

The State Inpatient Databases and State Emergency Department Databases from the Healthcare Cost and Utilization Project, sponsored by the Agency for Healthcare Research and Quality, were used for the states of California (2005–2011), Florida (2005–2014), and New York (2008–2014). Data on patients with proximal humeral fractures was extracted. Patients underwent non-operative or operative (ORIF or arthroplasty) treatment at baseline and were followed for at least 4 years from the index presentation. If the patient needed subsequent surgery, time to event was calculated in days, and Kaplan-Meier survival curves were plotted.

**Results:**

At the index visit, 90.3% of patients with proximal humeral fractures had non-operative treatment, 6.7% had ORIF, and 3.0% had arthroplasty. 7.6% of patients initially treated non-operatively, 6.6% initially treated with ORIF, and 7.2% initially treated with arthroplasty needed surgery during follow-up. Device complications were the primary reason for readmission in 5.3% of ORIF patients and 6.7% of arthroplasty patients (*p* < 0.0001). All-cause in-hospital mortality was 9.8% for patients managed non-operatively, 8.8% for ORIF, and 10.0% for arthroplasty (*p* = 0.003).

**Conclusions:**

A majority of patients with proximal humeral fractures underwent non-operative treatment. There was a relatively high all-cause in-hospital mortality irrespective of treatment. Given the recent debate on operative versus non-operative treatment for proximal humeral fractures, our study provides valuable information on the need for revision surgery after initial treatment. The differences in rates of revision surgery between patients treated non-operatively, with ORIF, and with arthroplasty were small in magnitude. At nine years of follow-up, ORIF had the lowest probability of needing follow-up surgery, and arthroplasty had the highest.

## Background

After the age of 50, the incidence of proximal humeral fractures increases before peaking between 80 to 89 years [1, 2]. Over 90% of these injuries are the result of a fall. Due to the growing elderly population, the number of fractures between 2008 and 2030 is expected to increase by 50% [1, 3]. Treatments for proximal humeral fractures include non-operative care and surgery. Surgical care includes open reduction and internal fixation (ORIF) and arthroplasty [4]. Some studies suggest that operative treatment of complex proximal humeral fractures, particularly in elderly patients, does not lead to a significantly improved outcome for patients as compared with non-operative treatment, and can lead to more complications and revision surgeries [5–9].

Few studies have used national databases to report the epidemiology of upper extremity procedures and specifically for humeral fractures [1, 10]. One study used state inpatient databases to track readmission rates after proximal humeral fracture for up to 90 days following operative treatments [11]. This study did not report on patients receiving non-operative treatment. To our knowledge, longitudinal data on complications, readmissions, and conversion of non-operative treatment to surgery or ORIF to arthroplasty has not been reported in large state or national datasets for proximal humeral fractures. Given the debate on best treatment approaches for proximal humeral fractures, this data provides insights into current practice patterns and failure of an initial treatment approach (as determined by conversion to a different surgical treatment). The aim of our study was to analyze practice patterns for patients with proximal humeral fractures in the states of California, Florida, and New York. We longitudinally followed patients for at least 4 years to report on complications and revision surgery.

## Methods

### Data sources

Data on patients with proximal humeral fractures was obtained from the Healthcare Cost and Utilization Project (HCUP), sponsored by the Agency for Healthcare Research and Quality (AHRQ) [12]. The State Inpatient Databases (SID) contain all or nearly all inpatient records from participating states [13]. Since patients with proximal humeral fractures may be discharged from the emergency room without being admitted to an in-patient facility, the State Emergency Department Databases (SEDD) that contain records of emergency department visits that do not result in admission were also used [14]. Information was obtained from these databases for patients in California whose index visit was between 2005 and 2007, and their follow-up visits were tracked through 2011 (last year for availability of California data); patients in Florida whose index visit was between 2005 and 2010, and their follow-up visits were tracked through 2014; and patients in New York whose index visit was between 2008 and 2010, and their follow-up visits were tracked through 2014. The states and years chosen were based upon the availability of longitudinal follow-up data to track an individual patient.

### Sample selection

Procedures and diagnoses were identified by coding from the International Classification of Diseases, Ninth Revision, Clinical Modification (ICD-9CM). Index visits were defined by any ICD-9-CM diagnosis code for closed fractures of the proximal humerus: 812.00, 812.01, 812.02, 812.03, or 812.09. Patients were excluded if their primary diagnostic code indicated that the surgery at their initial visit was for correction of a previous arthroplasty or complication, or if they had any diagnosis code for malignancies or pathological fractures of the humerus, scapula, or glenoid. Selected patients were then separated into three groups (non-operative, ORIF, and arthroplasty) depending on the treatment they received at their index visit. Any procedure code of 79.31 defined ORIF while 81.80 or 81.81 defined arthroplasty. Patients with a diagnosis code indicating a proximal humeral fracture but without a surgical procedure code were classified as receiving non-operative treatment.

### Comorbidities and outcome measures

Other information extracted from this initial visit included patient comorbidity, which was assessed with the Charlson Index (categorized into 0, 1, and ≥ 2), calculated from discharge diagnoses of the index visit, and modified for administrative data by Deyo [15, 16]. Disposition on discharge was classified as routine or non-routine (transfer to short-term hospital, skilled nursing facility, intermediate care facility, another type of facility, or home health care). Complications of the index admission included wound complications, pulmonary embolism, deep venous thrombosis (DVT) of lower extremity, venous embolism of thrombosis of other site, cardiac complications, cardiac rhythm conversion, and unspecified septicemia. The corresponding ICD-9CM diagnosis and procedure codes that were used to ascertain these complications are listed in Additional file 1. All-cause mortality was documented for all patients who died while in the hospital.

### Longitudinal follow-up

Each patient is provided a unique identifier in the databases that allows patients to be tracked over time. Patients were followed longitudinally for at least four years and up to nine years after the index visit to identify hospital readmissions. The primary reason for readmission was determined based on ICD-9 codes. The patient was determined to have a subsequent ORIF or arthroplasty if they had a corresponding ICD-9 procedure code at readmission.

### Statistical analysis

Characteristics of the patient population were analyzed as means, medians, standard deviations, and proportions. Chi-square tests and analysis of variance (ANOVA) tests were used to compare the three groups (non-operative, ORIF, and arthroplasty), and *p*-values were reported. Time to event (surgery or revision surgery) was calculated in days, and Kaplan-Meier survival curves were plotted [17].

## Results

A total of 134,411 patients were treated for a proximal humeral fracture in California (2005–2007), Florida (2005–2010), and New York (2008–2010) (Table 1). A majority of these patients were treated non-operatively (90.3%) while 6.7% underwent ORIF and 3.0% had an arthroplasty at the index visit. The mean age of patients undergoing non-operative treatment was 66 ± 22 years, ORIF was 65 ± 18 years, and arthroplasty was 74 ± 12 years (*p* < 0.0001). Approximately 70% of patients undergoing non-operative treatment and ORIF were female compared to 77.2% of those undergoing arthroplasty (*p* < 0.0001). The median length of hospital stay was 0 days for non-operative management, 4 days for ORIF, and 5 days for arthroplasty (p < 0.0001).

**Table 1:** Baseline Characteristics of Patients with Proximal Humeral Fractures in California, New York, and Florida

		Treatment			
	Total 134,411	Non-Operative 121,411 (90.3%)	ORIF 8994 (6.7%)	Arthroplasty 4006 (3.0%)	*p*-value^Π^
Age Mean ± SD; Median	65.8 ± 21.3; 71	65.7 ± 21.8; 71	65.0 ± 18.3; 68	73.5 ± 11.6; 76	< 0.0001
Female	94,342 (70.2%)	85,234 (70.2%)	6014 (66.9%)	3094 (77.2%)	< 0.0001
Charlson Index:					
0	93,182 (69.3%)	86,474 (71.2%)	4847 (53.9%)	1861 (46.5%)	< 0.0001
1	26,901 (20.0%)	22,998 (18.9%)	2560 (28.5%)	1343 (33.5%)	
≥ 2	14,328 (10.7%)	13,974 (11.5%)	1587 (17.6%)	802 (20.0%)	
Disposition on discharge:					
Routine	99,667 (74.2%)	94,643 (78.0%)	3887 (43.2%)	1137 (28.4%)	< 0.0001
Non-routine^a^	32,840 (24.4%)	25,014 (20.6%)	4995 (55.5%)	2831 (70.7%)	
Average hospital length of stay Mean ± SD; Median	2.0 ± 5.3; 0	1.5 ± 4.8; 0	6.1 ± 7.9; 4	6.2 ± 5.9; 5	< 0.0001
Number of chronic conditions^b^ Mean ± SD; Median	2.1 ± 2.6; 1	1.9 ± 2.5; 1	3.8 ± 2.8; 3	4.4 ± 2.7; 4	< 0.0001
Osteoporosis	8582 (6.4%)	6698 (5.5%)	1163 (12.9%)	721 (18.0%)	< 0.0001
Complications during in-hospital stay for index admission					
Wound complications	124 (0.09%)	N/A	31 (0.34%)	*	< 0.0001
Pulmonary embolism	221 (0.16%)	154 (0.13%)	42 (0.47%)	25 (0.62%)	0.06
Deep venous thrombosis of lower extremity	288 (0.21%)	225 (0.19%)	37 (0.41%)	26 (0.65%)	0.95
Venous embolism or thrombosis of other site	140 (0.10%)	97 (0.08%)	27 (0.30%)	16 (0.40%)	0.11
Cardiac complications	223 (0.17%)	110 (0.09%)	69 (0.77%)	44 (1.10%)	0.0003
Cardiac rhythm conversion	249 (0.19%)	208 (0.17%)	25 (0.28%)	16 (0.40%)	0.96
Unspecified septicemia	525 (0.39%)	458 (0.38%)	48 (0.53%)	19 (0.47%)	0.18

*ORIF* open reduction and internal fixation

*SD* standard deviation

* Value < 10

^Π^ *p*-values of non-operative vs. ORIF vs. arthroplasty as treatment

^a^Non-routine discharges include transfer to short-term hospital, skilled nursing facility, intermediate care facility, another type of facility, or home health care

^b^Includes data only from FL and NY

Patients undergoing ORIF were significantly more likely to have a wound complication as compared with patients undergoing arthroplasty (p < 0.0001) during their index admission (Table 1). There was also a significant difference in the proportion of patients with cardiac complications between those who received non-operative treatment versus ORIF versus arthroplasty (0.09% vs. 0.77% vs. 1.10%; *p* = 0.0003) during their index admission (Table 1). All-cause in-hospital mortality was 9.8% for patients managed with non-operative treatment, 8.8% for ORIF, and 10.0% for arthroplasty (*p* = 0.003; Table 2).

**Table 2:** Outcomes of Patients during Follow-Up

	Initial Treatment			
	Non-Operative 121,411	ORIF 8994	Arthroplasty 4006	*p*-value^Π^
Revision surgery^a^:				
ORIF	5410 (4.5%)	225 (2.5%)	73 (1.8%)	0.009
Arthroplasty	3747 (3.1%)	369 (4.1%)	217 (5.4%)	0.66
All-cause mortality while in-patient	11,876 (9.8%)	787 (8.8%)	401 (10.0%)	0.003
Reasons for readmission				
Device complications	N/A	475 (5.3%)	270 (6.7%)	< 0.0001
Wound complications	N/A	172 (1.9%)	73 (1.8%)	0.0002
Pulmonary embolism	30 (0.02%)	*	*	0.004
Deep venous thrombosis of lower extremity	1495 (1.2%)	130 (1.4%)	79 (2.0%)	0.33
Venous embolism or thrombosis of other site	127 (0.10%)	15 (0.17%)	*	0.22
Cardiac complications	60 (0.05%)	*	*	0.88
Cardiac rhythm conversion	2405 (2.0%)	150 (1.7%)	89 (2.2%)	0.02
Unspecified septicemia	7131 (5.9%)	491 (5.5%)	265 (6.6%)	0.01

*ORIF* open reduction and internal fixation

*SD* standard deviation

* Value < 10

^Π^ *p*-values of non-operative vs. ORIF vs. arthroplasty as treatment

^a^Percentages based on initial cohort of subjects

**Fig. 1:**
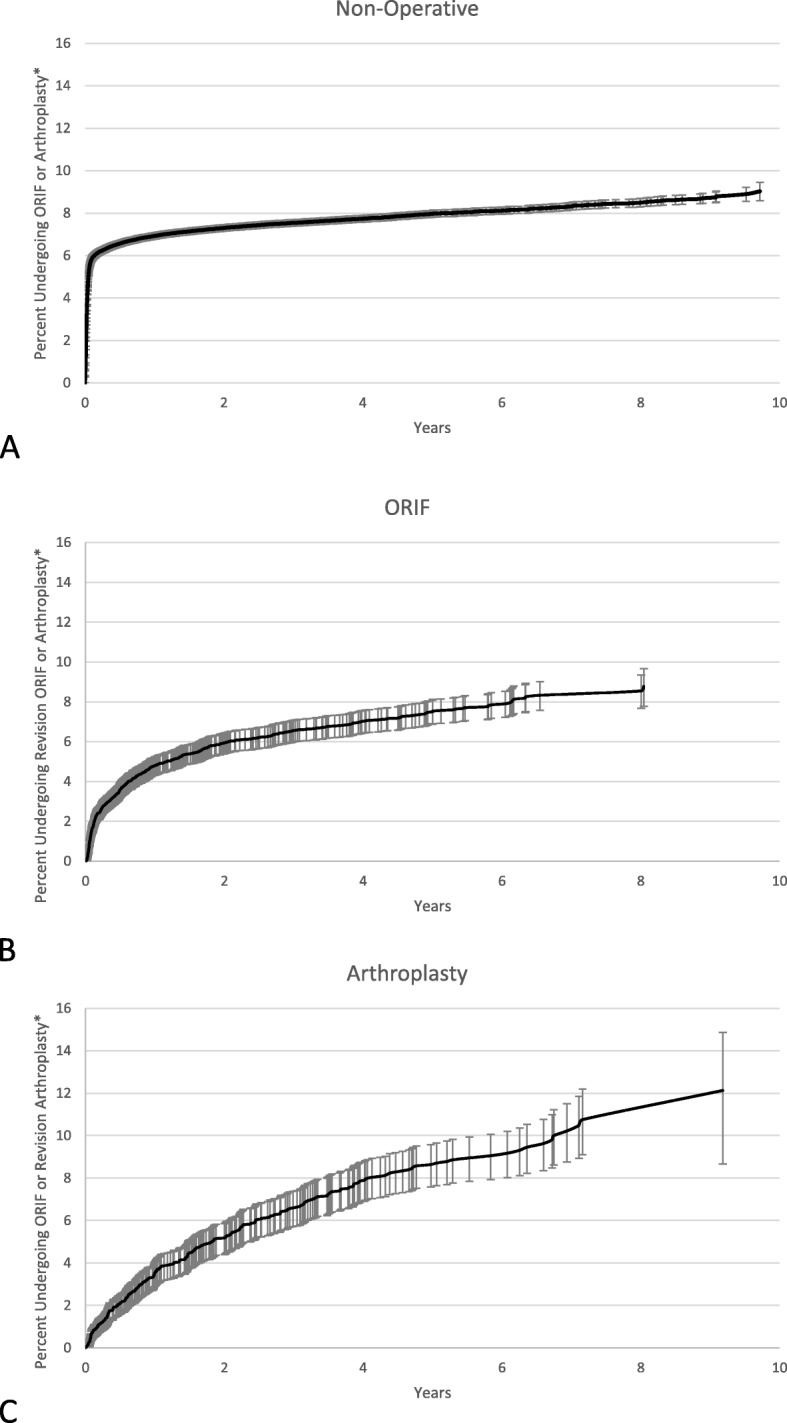
Kaplan-Meier survival curve of patients who initially underwent **a**) non-operative treatment, **b**) ORIF, and **c**) arthroplasty. * Percentages based on patients who had continued follow-up

Of the patients who had non-operative treatment initially, 4.5% required an ORIF and 3.1% underwent an arthroplasty during follow-up (Fig. 1a, Table 2). Subsequent arthroplasty was performed in 4.1% of the patients initially treated with ORIF and 5.4% of the patients initially treated with arthroplasty (Fig. 1b, c). Device complications were the primary reason for readmission in 5.3% of ORIF patients and 6.7% of arthroplasty patients (*p* < 0.0001).

There was variation in the time to readmission between states (Table 3). In California, the mean number of days to readmission for ORIF after initial non-operative treatment was 90.7 ± 284.3 days, after initial ORIF was 358.2 ± 527.5 days, and after initial arthroplasty was 482.9 ± 331.7 days (*p* < 0.0001). In Florida, the mean number of days to readmission for ORIF after initial non-operative treatment was 139.5 ± 436.0 days, after initial ORIF was 623.4 ± 739.2 days, and after initial arthroplasty was 1002.8 ± 791.5 days (*p* < 0.0001). In Florida, the mean number of days to readmission for arthroplasty after initial non-operative treatment was 192.9 ± 455.2 days, after initial ORIF was 378.6 ± 523.0 days, and after initial arthroplasty was 588.2 ± 628.9 days (p < 0.0001). In New York, the mean number of days to readmission for arthroplasty after initial non-operative treatment was 145.6 ± 341.6 days, after initial ORIF was 294.5 ± 345.3 days, and after initial arthroplasty was 568.0 ± 533.1 days (p < 0.0001).

**Table 3:** Time to Revision Surgery in Days

	Initial Treatment			
	Non-Operative	ORIF	Arthroplasty	*p*-value*
California:				
ORIF	90.7 ± 284.3; 1–2214	358.2 ± 527.5; 4–2209	482.9 ± 331.7; 14–1143	< 0.0001
Arthroplasty Mean ± SD; range	182.7 ± 375.9; 1–2279	317.1 ± 370.2; 9–1735	599.1 ± 487.3; 1–1847	< 0.0001
Florida:				
ORIF	139.5 ± 436.0; 1–3476	623.4 ± 739.2; 5–2938	1002.8 ± 791.5; 33–2616	< 0.0001
Arthroplasty Mean ± SD; range	192.9 ± 455.2; 1–3549	378.6 ± 523.0; 9–2392	588.2 ± 628.9; 9–3354	< 0.0001
New York:				
ORIF	89.8 ± 275.5; 1–2199	356.5 ± 491.8; 2–1827	606.0 ± 624.3; 23–1668	< 0.0001
Arthroplasty Mean ± SD; range	145.6 ± 341.6; 1–2330	294.5 ± 345.3; 6–1662	568.0 ± 533.1; 12–1551	< 0.0001

*ORIF* open reduction and internal fixation

*SD* standard deviation

* *p*-values of non-operative vs. ORIF vs. arthroplasty as treatment

## Discussion

We used large statewide in-patient and emergency room databases to assess management of patients with proximal humeral fractures. We also tracked this large cohort of patients in California, Florida, and New York for at least four years and up to nine years from index visit to report on readmissions and complications. We found that 90.3% of patients underwent non-operative treatment. The incidence of proximal humeral fractures was associated with an all-cause in-hospital mortality of 8.8–10.0%. Among patients undergoing ORIF, 5.3% of patients, and among those undergoing arthroplasty, 6.7% of patients required readmission due to device complications. Among patients undergoing initial non-operative treatment, 7.6% underwent subsequent ORIF or arthroplasty. Among patients undergoing initial ORIF, 6.6% required a revision ORIF or arthroplasty, and among patients undergoing initial arthroplasty, 7.2% required revision arthroplasty or ORIF during follow-up. The exact nature of the ORIF following arthroplasty is not known. Overall, arthroplasty had the lowest probability of survival (of procedure) at nine years of follow-up.

Zhang et al. reported that 3.1% of patients had surgical complications requiring readmissions using the HCUP State Inpatient Database for California (2005–2010), Florida (2005–2010), Hawaii (2006–2007), North Carolina (2006–2010), Nebraska (2006–2010), New York (2006–2009), and Utah (2006–2009) [11]. In our study, rates were slightly higher with 7.9% of patients having device and wound complications.

There is debate on operative versus non-operative treatment for proximal humeral fractures. Some previous studies have reported that patients undergoing operative treatment are more likely to have complications [6, 8, 9] and that no significant difference exists in outcomes after operative and non-operative treatment [5, 7]. Although our study was not designed to assess comparative-effectiveness of operative versus non-operative treatments for proximal humeral fractures, it provides data on complications, readmissions, in-hospital mortality, and time to revision surgery after non-operative treatment, ORIF, and arthroplasty. All-cause mortality during in-patient stay was lower for patients initially treated with ORIF (8.8%), followed by those treated non-operatively (9.8%) and with arthroplasty (10.0%). However, these differences were small in magnitude. It is possible that the lack of difference between in-hospital mortality after non-operative and operative treatment in our study represents a selection bias where patients who are unfit for surgery are frail at baseline. Previous studies have also reported that there was no significant difference in mortality between operative and non-operative treatments [5, 7]. Another study reported mortality rates at 24 months of 7.1% after non-operative treatment and 11.1% after hemiarthroplasty [18].

We report data on time to revision surgery after index treatment for proximal humeral fractures. This data is difficult to obtain from large epidemiological studies. In our study, patients who initially received ORIF needed revision ORIF or arthroplasty in 6.6% of cases, whereas those that initially underwent arthroplasty required revision surgery in 7.2% of cases. Patients initially undergoing non-operative treatment had subsequent ORIF or arthroplasty in 7.6% of cases. Previous studies have also found that initial operative treatment after proximal humeral fractures led to an increased number of additional surgeries during follow-up [5–7, 9]. In our study, the time to revision surgery was shortest for patients initially treated non-operatively followed by those who underwent ORIF and arthroplasty.

Limitations of our study include loss of follow-up of patients who received care in a different state from their index admission. We were unable to ascertain death in the community and hence only in-hospital mortality data is presented. Since administrative databases rely on ICD-9 billing codes, a coding error is possible leading to misclassification. However, we do not have evidence for differential misclassification by treatment group. Data from California was unavailable after 2011 hence longer term survival could not be determined in California. It is possible that in few cases, patients had surgery on the contralateral shoulder for proximal humeral fracture after the index admission. It is not possible to ascertain this from ICD-9 codes and could lead to overestimation of ORIF and arthroplasty procedures during follow-up. Despite these limitations, administrative databases are valuable for providing large-scale information on treatments for patients with proximal humeral fractures.

## Conclusions

This is a large study on patients with proximal humeral fractures in California, Florida, and New York. We report on patient characteristics, treatments, complications, and need for revision surgery. We found that a majority of patients underwent non-operative treatment. Although complications rates were low during the index hospital stay, 5.3% of patients undergoing ORIF and 6.7% of patients undergoing arthroplasty required readmission after discharge due to device complications. The incidence of proximal humeral fractures was associated with a relatively high all-cause in-hospital mortality irrespective of the treatment received. Given the recent debate on operative versus non-operative treatment for proximal humeral fractures, our study provides valuable information on the need for revision surgery after initial non-operative and operative treatment. The differences in rates of revision surgery between patients treated non-operatively, with ORIF, and with arthroplasty were small in magnitude. At nine years of follow-up, ORIF had the lowest probability of needing follow-up surgery, and arthroplasty had the highest.

## Supplementary information

**Additional file 1:** ICD-9CM diagnosis and procedure codes that were used to ascertain complications. (DOCX 12 kb)

## Data Availability

The data that support the findings of this study are available from the Healthcare Cost and Utilization Project (HCUP) sponsored by the Agency for Healthcare Research and Quality (AHRQ) but restrictions apply to the availability of these data, which were used under license for the current study, and so are not publicly available. Data are however available from the authors upon reasonable request and with permission of AHRQ.

## Abbreviations

AHRQ: Agency for Healthcare Research and Quality
ANOVA: Analysis of Variance
DVT: Deep Venous Thrombosis
HCUP: Healthcare Cost and Utilization Project
ICD-9CM: International Classification of Diseases, Ninth Revision, Clinical Modification
ORIF: Open Reduction and Internal Fixation
SEDD: State Emergency Department Databases
SID: State Inpatient Databases
