# Supplementary material for: Readmissions, revisions, and mortality after treatment for proximal humeral fractures in three large states
Source: BMC Musculoskelet Disord. 2019 Sep 11;20:419. doi: 10.1186/s12891-019-2812-9 (PMC6737688; doi:10.1186/s12891-019-2812-9)
Supplement: Supplementary file 1 — Additional file 1: ICD-9CM diagnosis and procedure codes that were used to ascertain complications. (DOCX 12 kb) [file 12891_2019_2812_MOESM1_ESM.docx]

Additional file 1

|  | **ICD-9 Diagnosis Code** |
| --- | --- |
| Closed proximal humeral fractures | 812.00, 812.01, 812.02, 812.03, 812.09 |
| Mechanical complication of internal orthopedic device, implant, and graft | 996.40, 996.41, 996.42, 996.43, 996.44, 996.45, 996.46, 996.47, 996.49 |
| Infection and inflammatory reaction due to internal joint prosthesis | 996.66 |
| Other complications due to internal joint prosthesis | 996.77 |
| Other complications due to internal orthopedic device, implant, and graft | 996.78 |
| Malignant neoplasm of scapula and long bones of upper limb | 170.4 |
| Pathologic fracture of humerus | 733.11 |
| Osteoporosis | 733.00, 733.01, 733.02, 733.03, 733.09 |
| Wound complications | 998.59, 999.3, 998.83, 998.3 |
| Pulmonary embolism | 415.19, 415.11 |
| Deep venous thrombosis of lower extremity | 453.40, 453.41, 453.42 |
| Venous embolism or thrombosis of other site | 453.8, 453.9 |
| Cardiac complications | 997.1 |
| Unspecified septicemia | 038.9 |

|  | **ICD-9 Procedure Code** |
| --- | --- |
| Arthroplasty | 81.80, 81.81 |
| Revision of arthroplasty | 81.97, 81.83 |
| ORIF | 79.31 |
| Conversion of cardiac rhythm | 99.60, 99.61, 99.62, 99.63, 99.64, 99.69 |
